# Optimisation of Dairy Soiled Water as a Novel Duckweed Growth Medium

**DOI:** 10.3390/plants14010110

**Published:** 2025-01-02

**Authors:** Cian Redmond, Neil E. Coughlan, Aine Purcell, Marcel A. K. Jansen

**Affiliations:** 1 School of Biological, Earth and Environmental Sciences, University College Cork, Distillery Fields, North Mall, T23 TK30 Cork, Ireland; 2 Environmental Research Institute, University College Cork, Lee Road, T23 XE10 Cork, Ireland

**Keywords:** remediation, valorisation, *Lemna minor*, nutrients, agricultural wastewater, circular economy

## Abstract

As a result of intensive agriculture, large quantities of liquid wastewaters are produced. Dairy soiled water (DSW) is produced in large volumes during the milking process of cattle. It comprises essential plant nutrients such as nitrogen, phosphorus, and potassium. The physicochemical properties of DSWs are highly variable as per individual farmer practices and seasonality. Currently, DSWs are disposed of primarily through land spreading, which frequently results in environmental pollution through land run-off. As a result of the large volumes produced coupled with the high variability, there are few alternative uses for DSWs, with presently little consideration of possible on-farm valorisation. Through a series of factorial experiments, the suitability of DSW as a novel duckweed (*Lemna minor* L.) cultivation medium is assessed. Different concentrations and pH values are assessed to determine the optimal conditions to support duckweed cultivation. Under the principles of circular economy, duckweed-based valorisation of DSWs can lead to the production of valuable, high-protein plant biomass that could be incorporated into animal feed to support livestock nutritional requirements. This study identifies the management of DSW’s pH as a key growth parameter in the successful cultivation of duckweed to underpin a circular economy approach for valorisation and improved environmental outcomes.

## 1. Introduction

A consequence of intensive agriculture is the generation of numerous liquid waste streams (e.g., slurries, digestates, and soiled waters), often with unique compositions depending on their origin and management [1]. These liquid wastes tend to be produced in large volumes that frequently require substantial and costly management and comprise regulatory and environmental burdens [2]. Many of these liquid wastes contain elevated concentrations of Biochemical and Chemical Oxygen Demand (BOD_5_ and COD, respectively) as well as nitrogen, phosphorus, and potassium, all of which represent a threat to freshwater ecosystems [3]. However, nitrogen, phosphorus, and potassium are also essential and valuable plant nutrients [4,5].

The primary mechanism for disposal of animal manures, slurries, and associated soiled wastewaters is through land spreading during the regulatory permitted season. This way, the waste stream is exploited as a source of nutrients, on which contemporary agriculture is highly dependent [6]. Yet, when excess quantities of animal waste are spread on land, it can run off into nearby water bodies, or nutrients can leach through soils, making their way into ground water systems, potentially polluting drinking water sources and damaging aquatic ecosystems. This effect is often aggravated by elevated levels of precipitation [7,8]. Furthermore, the storage and subsequent disposal of high-nutrient liquid wastes via land spreading contribute to global greenhouse gas emissions (GHG), namely ammonia (NH_3_), carbon dioxide (CO_2_), methane (CH_4_), and nitrous oxide (N_2_O) [9,10,11]. International policies such as the European Union (EU) Green Deal [12] and Farm to Fork [13] aim to create a more sustainable agricultural sector through legally binding targets on greenhouse gas emissions, namely, a 55% reduction by 2030. In turn, this will have substantial consequences for the management of various wastewaters.

One liquid waste that is produced in bulk on dairy farms is dairy soiled water (DSW). DSWs are produced during the milking process and in the subsequent rinsing and cleaning of the milking parlour and milking equipment [14]. It comprises cow faeces, urine, rinse water, detergents, sediment, rainwater, and runoff from concreted holding yards. Even modest-sized dairy farms produce in excess of 2000 litres DSW per day. Based on nitrogen, phosphorus, and potassium content, DSWs are generally considered a dilute liquid waste with low to modest fertiliser value. Thus, the issues related to DSW are substantially different from those of slurry. In 2023, Ireland’s Agriculture and Development Authority reported the fertiliser replacement value of DSWs was EUR 2.20 m^−3^, while slurry, which incurs the same spreading cost, was valued significantly higher at EUR 9.70 m^−3^ [15]. Yet, DSWs carry considerable costs related to storage and disposal [16], which can negatively impact industry compliance at times. In effect, this cost is considered disproportionate, given the limited fertiliser value of this wastewater. DSWs are also less suitable for large-scale anaerobic digestion due to relatively low solid contents and unfavourable carbon to nitrogen ratio, which would lead to a low output of biogas [17]. Conversely, there is a growing volume of DSW at farms linked to the implementation of new regulations concerning run-off from milking parlours, yards, and roadways. Thus, innovative solutions are required to remediate and/or valorise this emerging liquid waste stream.

There is substantial scope to develop novel, alternative, and innovative uses to valorise liquid waste streams such as, for example, DSWs. Developing alternative uses for DSWs is in line with the principles of circular economy and can contribute to the transition to more sustainable global food systems that build resilience both to climate change and to changing consumer demands [18,19]. In the present study, the suitability of DSW as a novel growth medium for duckweed (*Lemna minor* L.) cultivation is explored. Duckweeds (*Lemnaceae* spp.) are small floating aquatic plants known for their rapid growth and high rate of nutrient uptake, particularly for nitrogen and phosphorus [20,21]. Duckweed’s ability to grow on liquid wastes is well documented, including diluted animal slurries (e.g., [4,22,23]), although DSW as produced by dairy milking parlours has not previously been considered. Duckweed species tolerate, and in some cases prefer, ammonium, making them ideal candidates for cultivation on DSWs [24]. Many studies have demonstrated duckweed’s rapid ability to uptake nitrogen and phosphorus in large quantities, achieving uptake rates of 547 mg m^−2^ and 200 mg m^−2^, respectively [25,26]. Duckweed-based wastewater treatment has also been shown to significantly decrease the BOD_5_ and Chemical Oxygen Demand (COD) of wastewaters [27]. Previously, the suitability of duckweed-based cultivation within the agricultural industry (e.g., aquaculture) has highlighted the potential for the production of a high protein plant biomass with simultaneous cleaning of wastewater [28].

Furthermore, the generated duckweed biomass can potentially serve as a sustainable protein source, subject to phytosanitary considerations. Duckweed protein has a similar amino acid profile to that of soybean [29] and can have a dry-weight protein content of 30–45% under optimal conditions [30]. The suitability of duckweed as a substitute for soybean meal in animal feed has been demonstrated by numerous studies [31,32]. By incorporating duckweed as part of animal feed, nitrogen and phosphorus can be retained in the food chain, thus closing the nutrient loop [33]. Furthermore, duckweed-based wastewater valorisation can potentially address the escalated demand for plant-based and other non-animal protein sources in a sustainable and environmentally friendly manner [34].

In the present study, for the first time, milking-parlour-generated and tank-stored DSWs, which are typical of dairy farm management practices, were assessed for duckweed cultivation. In doing so, we (1) explore the suitability of DSWs as a medium for duckweed cultivation and (2) optimise duckweed growth on DSWs.

## 2. Results

### 2.1. Dairy Soiled Water Physicochemical Properties

The physicochemical properties of five dairy soiled water (DSW) samples are detailed in Table 1. Also shown are mean values for 60 Irish dairy farms measured over a 12-month period [14]. These data show a large variation in nutrient composition among samples collected as part of the present study. DSWs dry matter content ranged from 0.23 to 1.13%, and the BOD_5_ concentration ranged from 939 to 6521 mg L^−1^, with only one sample (Sample 2) above the limit of 2500 mg L^−1^. Total nitrogen varied from 164 to 1250 mg L^−1^. Total ammoniacal nitrogen represented most of the total nitrogen in the medium, ranging from 108 to 871 mg L^−1^. Total phosphorus ranged from 24 to 134 mg L^−1^, comprising mostly orthophosphate, with concentrations ranging from 23 to 68 mg L^−1^. Potassium concentrations in collected samples ranged from 129 to 1350 mg L^−1^.

### 2.2. Cultivation of L. minor on Dairy Soiled Water with Matched Total Nitrogen Concentrations

**Figure 1:**
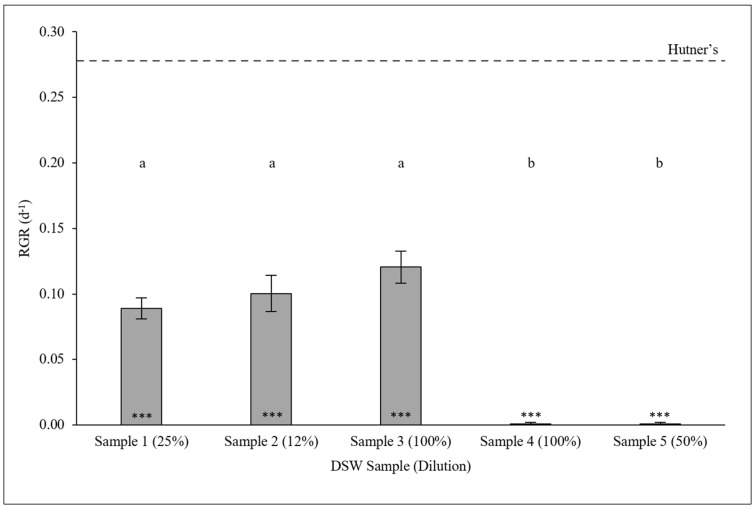
Mean RGR (±standard error [SE]) of *Lemna minor* grown on dairy soiled water sourced from different farms and diluted to achieve similar total nitrogen concentrations at pH 6 (*n =* 3). Mean RGR for control samples on half-strength Hutner’s is depicted by the dashed line (*n =* 3). Shared letters denote statistical similarity (*p* > 0.05), while star symbols represent significant differences to the half-strength Hutner’s control medium (***; *p* < 0.001).

The relative growth rate (RGR) of *L. minor* grown on the assessed DSW samples, diluted to similar total nitrogen concentration (150—189 mg L^−1^ N), significantly differed (GLM: χ^2^ = 71.183, df = 5; *p* < 0.0001; Figure 1). Post hoc analysis revealed significant differences among *L. minor* grown on DSW samples compared with the control grown on half-strength Hutner’s medium (all *p* < 0.001; Figure 1) [35]. Overall, duckweed grown on DSW did show lower RGR values compared with controls. In turn, the dilution matched DSW Samples 1, 2, and 3 did not differ from each other (i.e., *p* > 0.05), nor did Samples 4 and 5. However, Samples 1–3 significantly differed from both Samples 4 and 5 (all *p* < 0.001), as the latter two samples did not yield duckweed growth (Figure 1).

### 2.3. Cultivation of L. minor on Dairy Soiled Water of Differing Concentrations and Varied pH Values

**Figure 2:**
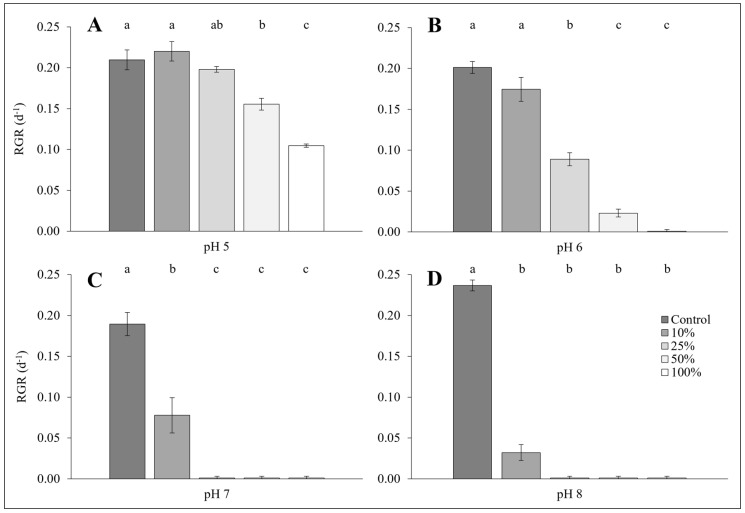
Mean RGR (±SE) of *Lemna minor* grown on four concentrations of dairy soiled water (Sample 1: 10%, 25%, 50%, and 100%), along with a half-strength Hutner’s control, at four pH values (5, 6, 7, and 8) (*n =* 3). Shared letters within each panel (**A**–**D**) denote statistical similarity (*p* > 0.05).

The RGR of *L. minor* grown on various concentrations of DSW and at several pH values significantly differed among treatments (GLM: χ^2^ = 240.28, df = 19; *p* < 0.0001; Figure 2). Higher DSW concentrations were found to significantly decrease *L. minor* RGR, as did increased pH (both *p* < 0.0001). In turn, an interactive effect of increased concentration and pH was also shown to reduce duckweed RGR on DSW (*p* < 0.0001). At pH 5, duckweed grew at all DSW concentrations (Figure 2A), with growth reducing as the DSW concentration increased. At pH 6, *L. minor* growth greatly decreased with increasing concentration, with no growth achieved at a 100% DSW concentration (Figure 2B). Only duckweed grown at a 10% concentration survived on DSW at pH 7 (Figure 2C), while growth was severely reduced or unsuccessful on DSW at pH 8 across all concentrations (Figure 2D). Post hoc analysis shows significantly different RGRs among tested concentrations at each pH value (see Figure 2).

### 2.4. Cultivation of L. minor on Unfiltered, Filtered, or Synthetic Dairy Soiled Water

**Figure 3:**
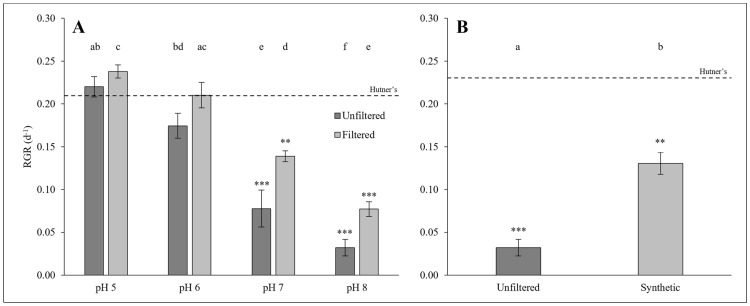
(**A**) Mean RGR (±SE) of *Lemna minor* grown on 10% unfiltered and filtered dairy soiled water (Sample 1) at different pH values (*n =* 3). (**B**) Mean RGR (±SE) of *Lemna minor* grown on 10% unfiltered dairy soiled water (Sample 1) compared with 10% dilution of synthetic dairy soiled water, both at pH 8 (*n =* 3). Mean RGR for half-strength Hutner’s control medium is depicted by the dashed line. Shared letters denote statistical similarity (*p* > 0.05) within each panel, while star symbols represent significant differences to the control (** *p* < 0.01, *** *p* < 0.001).

RGRs for *L. minor* grown on unfiltered or filtered DSW, at various pH values, were found to differ significantly among treatments (GLM: χ^2^ = 63.551, df = 4; *p* < 0.0001; Figure 3A). The RGR significantly increased on a 10% dilution of coarsely filtered DSW (*p* < 0.0001), while an increased pH significantly decreased RGR (*p* < 0.0001). Post hoc analysis shows significantly different RGRs among tested treatments, but no interactive effect was detected among the factors of filtration status or pH level (see Figure 3A).

The RGR of *L. minor* grown on unfiltered 10% DSW at pH 8, synthetic 10% DSW at pH 8, and the half-strength Hutner’s control medium differed significantly (GLM: χ^2^ = 24.46, df = 2; *p* < 0.0001; Figure 3B). The RGR for duckweed grown on the unfiltered 10% DSW sample was significantly lower than the RGR obtained for *L. minor* cultivated on the 10% synthetic DSW sample (*p* < 0.01; Figure 3B). In turn, the RGRs of unfiltered and synthetic-media-grown duckweed significantly differed from the control RGR (*p* < 0.001 and *p* < 0.01, respectively; Figure 3B).

### 2.5. Ammonium and Un-Ionised Ammonia Present in Dairy Soiled Water

**Figure 4:**
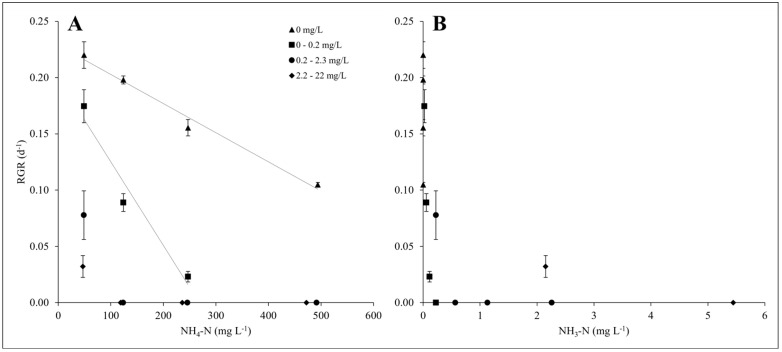
(**A**) Calculated ammonium (NH_4_-N) concentrations present in different dilutions of dairy soiled water (DSW) (Sample 1) and their relationship with the mean (±SE) *Lemna minor* RGR (*n =* 3). The insert gives the concentration of un-ionised ammonia (NH_3_-N) also present in the DSW. Linear relationships were detected between ammonium concentration and RGR, which were determined by the concentrations of un-ionised ammonia present: 0 mg L^−1^ NH_3_-N: *y* = 0.2287–0.0003*x*, *R*^2^ = 0.9846; 0–0.2 mg L^−1^ NH_3_-N: *y* = 0.1994–0.0007*x*, *R*^2^ = 0.9549 (Figure 4A). (**B**) Shows the relationship between calculated un-ionised ammonia concentrations and mean (±SE) *L. minor* RGR in DSW (Sample 1) dilutions (*n =* 3).

It was explored to what extent un-ionised ammonia was associated with decreased duckweed growth at higher pH values. Based on the concentration of total ammoniacal nitrogen of Sample 1 (Table 1), ammonium (NH_4_-N) and un-ionised ammonia (NH_3_-N) concentrations were calculated at different pH values according to [36]. It was observed that RGR decreased with increasing ammonium and un-ionised ammonia concentrations (Figure 4). Where un-ionised ammonia was absent, concentrations as high as 400 to 500 mg L^−1^ NH_4_-N did still facilitate some *L. minor* growth. In comparison, concentrations less than 1 mg L^−1^ NH_3_-N were linked to no growth (Figure 4). A linear relationship was found between *L. minor* RGR and ammonium concentrations (Figure 4) when un-ionised ammonia was absent (*R*^2^ = 0.9846) and when un-ionised ammonia concentrations were between 0–0.2 mg L^−1^ NH_3_-N (*R*^2^ = 0.9549).

### 2.6. Cultivation of L. minor on Dairy Soiled Water with Either pH Adjustment on Day 0-Only or Daily

**Figure 5:**
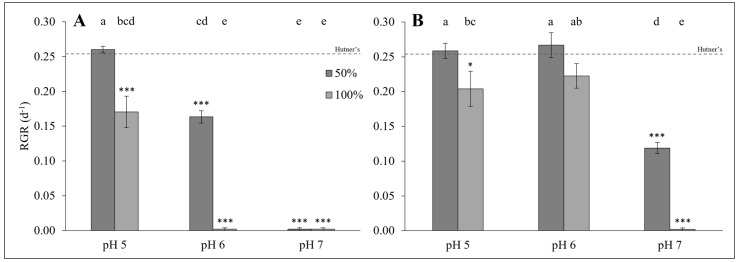
(**A**) Mean RGR (±SE) of *Lemna minor* grown on dairy soiled water (Sample 4: 50% and 100%) with pH set on Day 0 only (*n =* 5). (**B**) Mean RGR (±SE) of *Lemna minor* grown on dairy soiled water (Sample 4: 50% and 100%) with pH adjusted daily for seven days (*n =* 5). Mean RGR for control samples on half-strength Hutner’s medium is depicted by the dashed line. Shared letters denote statistical similarity (*p* > 0.05) within each panel, while star symbols represent significant differences to the control (* *p* < 0.05, *** *p* < 0.001).

RGRs of *L. minor* differed significantly depending on whether plants were grown on DSW, of which the pH values were adjusted on Day 0 only or on a daily basis over the duration of the experiment (GLM: χ^2^ = 239.24, df = 12; *p* < 0.0001; Figure 5). The set pH value, increased DSW concentration, and type of pH adjustment (i.e., Day 0-only or daily) all significantly affected duckweed RGR (all *p* < 0.0001; Figure 5). In addition, an interactive effect of increased pH and DSW concentration also had a significant negative effect on duckweed RGR (*p* < 0.05). Finally, a significant three-way combination effect of the pH value, type of pH adjustment, and DSW concentration on duckweed RGR was also detected (*p* < 0.0001). Post hoc analysis confirmed a significant increase in *L. minor* RGR on DSW where the pH was maintained daily when compared with when the pH was set on Day 0 only (pH 6), across both a 50% and 100% concentration (all *p* < 0.0001; Figure 5). Similarly, there was a significant increase in *L. minor* RGR at pH 7 when the pH was maintained daily compared with when the pH was set on Day 0 only (*p* < 0.0001; Figure 5).

### 2.7. Changes in Dairy Soiled Water pH

**Figure 6:**
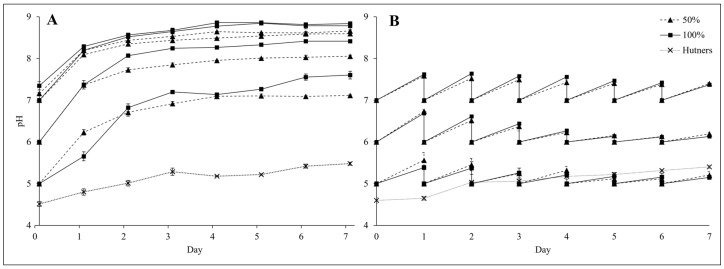
(**A**) Mean pH (±SE) in dairy soiled water (Sample 4; 50% and 100%) over a seven-day period with the pH set on Day 0 only (5, 6, 7, and the natural unamended pH) (*n =* 5, except Days 4 and 5 where *n =* 2). (**B**) Mean pH (±SE) of dairy soiled water (Sample 4; 50% and 100%) when the pH was adjusted daily (5, 6, 7 pH) (*n =* 5). An unadjusted half-strength Hutner’s control was used in both cases.

During experiments, pH values increased. The change in pH of 50% and 100% DSW, as well as of the control medium, half-strength Hutner’s, was recorded daily for the duration of the experiment (Figure 6). For samples with pH adjusted on Day 0 only, the pH rapidly increased from Day 0 to Day 3, after which it continued to increase at a lower rate and, in some cases, stabilised (Figure 6A). Changes in pH values for DSW that received a daily pH adjustment were apparent but tended to be less pronounced than those observed for non-adjusted samples (Figure 6). Daily adjusted samples also appeared to stabilise from Day 4 onwards (Figure 6B).

## 3. Discussion

The present study confirms that the physicochemical properties of dairy solid waters (DSWs) are highly variable across individual farms and seasons. Minogue et al. [14] identified farmer practices that are responsible for some of this variation. Increases in milking duration, area of the yard that receives rainfall, and area rinsed all led to a higher production of DSWs per cow [14]. Local climate can further influence DSWs’ physicochemical properties, with higher precipitation associated with a greater volume produced and lower concentration of nutrients. Increased milking duration, cows per milking unit, and parlour scraping frequency led to increased concentrations of total nitrogen, ammoniacal nitrogen, potassium, dry matter, and biochemical oxygen demand (BOD_5_) [14]. The high variability and volume produced of DSWs present a challenge to the development of alternative uses or the treatment of DSWs.

A comparison of the physicochemical properties of DSWs with the concentrations required for duckweed growth [37] suggests that duckweed might be successfully cultivated on DSWs. For example, concentrations of total nitrogen, total phosphorus, and potassium are all within the maximum tolerated by duckweed. Also, the calcium to magnesium ratio in all DSW samples should support duckweed growth [37]. DSWs contain essential trace elements for duckweed growth (i.e., zinc, copper, and iron; Table 1), with none of the tested trace elements being greater than the maximum tolerated by duckweed [37]. Thus, based on nutrient content, DSWs appear to be a suitable growth medium for duckweed.

The data show that ammoniacal nitrogen is the primary form of nitrogen in DSWs. Successful duckweed cultivation on liquid wastes, similar to DSWs, where the primary nitrogen source is ammoniacal nitrogen, is well documented in the literature [38,39]. In fact, duckweed species preferentially take up ammonium over nitrate [24,40]. While it is noted that the physicochemical properties of DSWs are variable, this might not necessarily be a major problem. Paolacci et al. [41] have previously demonstrated the ability of two duckweed species, including *L. minor*, to grow on a wide range of different nutrient concentrations. Thus, it was hypothesised that duckweed cultivation might be a suitable alternative use for DSWs, reducing the volume to spread for farmers while simultaneously recovering valuable nutrients and providing high-quality plant biomass that is high in protein.

Despite the hypothesised suitability of DSWs for duckweed cultivation, the growth of *L. minor* on unamended DSWs was poor to modest. This can be speculated to be due to several factors. Nutrient deficiencies can slow growth. However, analysis of the elemental composition of DSWs does not directly indicate any major deficiencies that would inhibit duckweed growth. Moreover, duckweed fronds can often grow for considerable periods of time on nutrients present in the parent fronds before the growth rate slows [41]. Thus, deficiencies are unlikely to explain observed low duckweed RGR values on DSWs. Toxic substances can also impede growth. Physicochemical analysis did not yield evidence of toxic constituents in DSWs, which might cause toxicity leading to duckweed mortality. Upon testing, the pH of DSWs was not deemed to be outside of the range for duckweed growth [42]. The growth of duckweed can also be hampered by strong microbial or algal growth in the medium [43,44]. Microbial growth was observed and was not unexpected given the non-sterile character of the DSW samples, as well as the presence of substantial amounts of suspended solids. DSWs contain up to 1% dry matter [14]. Furthermore, microbial growth can potentially alter the composition of the medium (e.g., by altering the pH and nutrient availability), resulting in indirect toxicities [43,45,46]. Thus, microbial growth on suspended solids is a possible explanation for slow duckweed RGR on DSWs.

To determine if the growth of *L. minor* on DSW was inhibited by suspended solids and subsequent microbial growth, suspended solids were partially removed from DSW using a filtration approach. In this study, it was found that centrifugation coupled with coarse filtration of DSW provided some benefits in terms of improved duckweed growth rates. Yet, a synthetic DSW comprised of inorganic salts only facilitated good duckweed growth, suggesting that suspended organic materials in DSWs contribute to duckweed growth impairment. Thus, a filtration approach can contribute to the improved growth of *L. minor* on DSWs. Separation of solids from liquid in animal slurries is a common agricultural practice [47]. The liquid fraction contains bioavailable nutrients, such as ammoniacal nitrogen and other soluble nutrients, whereas the solid fraction contains organic matter and phosphorus, which requires further steps to degrade into bioavailable nutrients [48,49]. In practice, nature-based solutions such as constructed wetlands are often utilised in the treatment of agricultural waste streams such as DSWs. Constructed wetlands have a series of ponds, with the first pond often acting as a settling pond for suspended solids. Sand filtration beds can also be used to separate solids from liquids. Other wastewater treatment methods make use of chemical additives such as flocculants and coagulants to bind the suspended solids and precipitate them [50,51,52].

Microbial and/or algal growth can contribute to a rise in the pH of DSWs [46,53,54,55,56]. In turn, this may affect the formation of un-ionised ammonia (NH_3_-N). The primary form of nitrogen in DSWs is ammoniacal nitrogen. Ammoniacal nitrogen comprises ammonium (NH_4_-N) and un-ionised ammonia (NH_3_-N). The equilibrium between ammonium and un-ionised ammonia is temperature- and pH-dependent [36]. Low concentrations of un-ionised ammonia are highly toxic to aquatic organisms [57]. Concentrations above 8 mg L^−1^ NH_3_-N have been shown to cause duckweed mortality, while lower concentrations lead to the inhibition of growth [58,59,60,61]. The present study shows that low (<0.03 mg L^−1^ NH_3-_N) estimated concentrations of un-ionised ammonia in medium set at specific pH values (see Figure 4) are associated with a marked slowdown of duckweed growth. To explain this marked sensitivity to un-ionised ammonia, the dynamic character of the DSW pH was analysed in more detail. It was noted that the pH of DSWs rapidly rises with time, an effect that may be associated with both microbial [56] as well as duckweed [24] growth. Thus, by using the pH set at Day 0 to calculate the concentration of un-ionised ammonia in DSWs, the concentration of un-ionised ammonia concentrations at later time points will have been underestimated. For example, the pH of DSW Sample 4 increased from a set value of pH 6 to pH 8 in just two days, and this would have increased the concentration of un-ionised ammonia from 0.07 mg L^−1^ to 6.41 mg L^−1^ NH_3_-N. In turn, this concentration of un-ionised ammonia is associated with significant inhibition of duckweed growth [58,60]. Therefore, it was hypothesised that by preventing the rise in pH, toxicity by un-ionised ammonia can be avoided, and DSWs would be a suitable substrate for duckweed cultivation. Consistently, this study shows for the first time that daily pH management of DSWs turns this wastewater into a suitable substrate for duckweed cultivation, opening a new perspective on wastewater remediation and valorisation.

A key conclusion of this study is that frequent monitoring and adjusting the pH of DSWs are critical for duckweed cultivation, valorisation, and remediation. Acidification of slurry on farms is a method already employed to reduce greenhouse gas emissions and reduce nitrogen losses [9,62,63]. Furthermore, dairy farms regularly use phosphoric acid to clean milking lines and equipment. Therefore, the idea of acidifying DSW is realistic in agricultural settings.

There are several additional benefits to acidifying DSWs. Acidifying slurry, and other ammoniacal-nitrogen-rich waste streams, reduces ammonia volatilisation [9,64]. By reducing ammonia emissions, nitrogen is retained, and potentially nitrogen use efficiency is increased, which reduces dependence on synthetic fertilisers. Furthermore, ammonia retention in DSWs avoids potentially negative environmental impacts. Atmospheric ammonia is often deposited a short distance from its emission point; this leads to the deposition of excess nitrogen in terrestrial and aquatic ecosystems, causing eutrophication [65]. Ammonia can also contribute to the formation of PM_2.5_ fine particulate matter, which poses a serious threat to human health. In 2018, the global agricultural sector was responsible for over 87% of ammonia emissions, with over one-quarter of agriculture’s emissions coming directly from manure management [66]. Thus, DSWs pH management enables duckweed cultivation while also reducing emissions which benefits the wider community.

## 4. Materials and Methods

### 4.1. Dairy Soiled Water Collection

Five samples of dairy soiled waters (DSWs) were collected from four dairy farms (labelled samples 1–5) in Co. Cork, Ireland, between July 2022 and October 2023. Samples 3 and 4 were collected from the same farm on separate occasions. The samples were collected from designated underground collection tanks at each farm. These tanks only received washings from the milking parlour, as well as the adjacent pre- and post-milking concreted holding yards. Tanks were not agitated prior to collection. On each sampling occasion, a minimum of 50 L of sample was collected using clean 25 L drums. Samples were transported to the laboratory and stored in a temperature-controlled room (4 ± 1 °C).

### 4.2. Duckweed Cultivation

The duckweed species used for this study was *Lemna minor* L. ‘Blarney’ (strain I.D. 5500: Rutgers Duckweed Stock Cooperative). Axenic stock cultures were cultivated in a controlled growth room with an average light intensity of 50 ± 5 µmol m^−2^ s^−1^ and a 16:8 light/dark photoperiod at a temperature of 22 ± 2 °C. Plants were cultivated on modified half-strength Hutner’s medium [35], an optimised growth medium that facilitates good growth of *L. minor* as described by Walsh et al. [37] for a minimum of two weeks prior to use in experiments.

### 4.3. Sequence of Experiments

Initially, *L. minor* was grown on DSWs sourced from different farms and diluted to achieve similar total nitrogen concentrations (150–189 mg L^−1^ N). DSW samples used in initial experiments were adjusted to pH 6 on Day 0. In a subsequent experiment, *L. minor* was grown on four concentrations of DSW (Sample 1: 10%, 25%, 50%, and 100%) and half-strength Hutner’s medium at four pH values (pH 5, 6, 7, and 8).

To study the effects of suspended solids present in DSWs, *L. minor* growth was compared across a 10% concentration of unfiltered or filtered DSW (Sample 1) at different pH values (5, 6, 7, and 8 pH). This was complimented with an assessment of *L. minor* growth on 10% unfiltered DSW (Sample 1, pH 8) compared with a 10% dilution of synthetic DSW at pH 8.

Finally, the effects of one-off and daily pH adjustment (pH 5, 6, and 7) on growth were studied using two different dilutions (50% and 100%) of DSW (Sample 4).

### 4.4. Experimental Design

All experiments were undertaken in Magenta vessels (Magenta GA-7 Plant Culture Box) with a final volume of 100 mL and lasted for seven days, as detailed before [23,37]. Experiments were conducted in a controlled growth room with an average light intensity of 50 ± 5 μmol m^−2^ s^−1^ and a 14:10 light/dark photoperiod at a temperature of 22 ± 2 °C. All experiments were conducted at least in triplicate.

Experiments commenced on Day 0 with three four-frond colonies taken from an axenic stock culture and placed in each replicate. To determine initial fresh biomass, *L. minor* colonies were patted dry using absorbent paper towel and then weighed using a fine balance (Fisherbrand Analytical Series). The medium temperature and pH were measured using a benchtop pH meter (Orion Star A111, Fisherbrand). On the final day of experiments (Day 7), *L. minor* was weighed using the same process for measuring initial biomass, and pH and temperature were measured and recorded.

### 4.5. Plant Parameters

Relative Growth Rate (RGR) was calculated using fresh biomass measurements with the following equation [67]:

$$RGR=\frac{\ln\left(\frac{W_{2}}{W_{1}}\right)}{\Delta T}$$

where *ln* is the natural logarithm, *W*_1_ is the initial fresh weight (Day 0), *W*_2_ is the final fresh weight (Day 7), and *ΔT* is the duration of the experiment (7 days).

### 4.6. Preparation of Dairy Soiled Water Samples

A full physicochemical analysis was immediately conducted for each freshly collected DSW sample by an accredited testing laboratory (INAB EN ISO/IEC 17025; ALS Life Sciences Ltd., Clonmel, Co. Tipperary, Ireland). Unfiltered samples were analysed to determine total solids, total phosphorus, pH, chemical oxygen demand, biochemical oxygen demand (BOD_5_), and total nitrogen using standard methods [68]. For determination of sulphate, ammoniacal nitrogen, nitrate, nitrite, total oxidised nitrogen, orthophosphate, chloride, and trace metals concentrations samples were filtered (0.45 μm) prior to analysis using standard methods [68].

Prior to experimental use, DSW samples were agitated by hand shaking the 25 L sample drums for 90 s to ensure mixing and sample homogeneity. Where indicated, DSWs were centrifuged in 50 mL conical centrifuge tubes (Thermo Scientific, Waltham, MA, USA) at 2800× *g* for five minutes. The supernatant was then passed through a cellulose filter assisted by a vacuum pump, which retained particles > 20 μm. Samples were diluted with deionised water to desired concentration.

Based on the physicochemical properties of Sample 1 (see Table 1), a synthetic DSW medium was developed using inorganic salts with the final composition shown in Table 2. The composition of the synthetic medium was representative of the dissolved nutrients in DSW Sample 1 but without the presence of suspended solids or organic matter. The pH of the synthetic medium was adjusted to pH 8 using 1M NaOH.

### 4.7. pH Monitoring and Adjustment

To monitor the change in pH of DSW samples used as duckweed growth medium, the pH was set at the start of the experiments (i.e., Day 0) at pH 5, 6, 7, and the natural unamended pH. Where indicated, the pH was altered using either 1M HCl or 1M NaOH on Day 0 only. In turn, the pH was measured again on Day 1, Day 2, Day 3, Day 4, Day 5, Day 6 within the overall duration of the experiment (Day 0–Day 7) using a benchtop pH meter. In experiments where the pH of the medium was kept constant, the pH was recorded daily for seven days and adjusted at 24 h intervals. For experiments where the pH was adjusted daily, the pH was recorded prior to adjusting the pH using HCl or NaOH.

The concentration of un-ionised ammonia present in DSWs on Day 0 was calculated using Emerson et al. [36]. In short, the un-ionised ammonia concentration was calculated as a fraction of the total ammoniacal nitrogen (Table 1), considering the dilution of DSW, the pH, and the temperature.

### 4.8. Data Analysis

Statistical analyses were conducted using R software (version R4.2.2). All data were assessed for normality of residual distributions (Shapiro–Wilk test: library *psych*) and homoscedasticity of variances (Levene’s test: library *car*). Where data were found to be normally distributed (*p* > 0.05) with homoscedastic residuals (*p* > 0.05), general linear models (analysis of variance [ANOVA]) were used to analyse differences in RGR. Logistic regression in the form of generalised linear models (GLM: *car*) was employed for non-normal data and/or heteroscedastic residuals (*p* < 0.05). A stepwise depletion approach was used to remove non-significant terms if required. Overall model significance was determined using likelihood ratio tests in all cases (library *lmtest*). Where *p*-values were significant (α < 0.05), a Tukey adjustment for the multiple pairwise comparison was used for post hoc analysis (library *emmeans*).

## 5. Conclusions

This study emphasises that DSWs present an opportunity for innovative valorisation. Under the principles of circular economy, DSWs can become a valuable resource for duckweed cultivation, simultaneously producing a protein-rich plant biomass whilst recovering valuable nutrients from DSWs. This study identifies the pH as the most critical growth parameter to monitor and adjust in order to successfully cultivate duckweed on DSWs. It is concluded that careful pH management has benefits for duckweed cultivation, as well as for nutrient retention and the wider environment. Future studies should focus on determining the potential of the phytoremediation capacity, the biomass yield, and the composition of duckweed cultivated on DSWs in upscaled or in situ systems.

## Figures and Tables

**Table 1:** Physicochemical properties of dairy soiled water samples, compared with the mean values (±standard deviation).

Parameter	Sample 1		Sample 2		Sample 3		Sample 4		Sample 5		Irish Mean ^3^ ± SD *	
	mg L^−1^	mM	mg L^−1^	mM	mg L^−1^	mM	mg L^−1^	mM	mg L^−1^	mM	mg L^−1^	mM
Total Solids	10,980	-	11,316	-	2956	-	2260	-	4852	-	5000 ± 5200	-
BOD_5_	2206	-	6521	-	1501	-	939	-	1865	-	2246 ± 2112	-
COD	11,530	-	16,000	-	3540	-	2380	-	4950	-	n.d	n.d
Total Nitrogen—N	756	54	1250	89.3	164	11.7	187	13.4	316	22.6	587 ± 536	41.9 ± 38.3
TON—N	<0.25	<0.02	<0.25	<0.02	<0.25	<0.02	<0.25	<0.02	<0.25	<0.02	n.d	n.d
Ammonia—N	494	35.3	871	62.2	108	7.7	146	10.4	228	16.2	212 ± 206	15.1 ± 14.7
Total Phosphorus—P	116	3.7	134	4.3	51	1.6	24	0.8	58	1.9	80 ± 68	2.6 ± 2.2
Phosphate—P	67 ^1^	2.2	68 ^1^	2.2	46 ^1^	1.5	23 ^1^	0.7	49 ^1^	1.6	36 ± 53 ^2^	1.2 ± 1.7
Sulphate—SO_4_^2−^	1962	20.4	1584	16.5	464	4.8	328	3.4	586	6.1	n.d	n.d
Chloride	1151	32.5	992	28	171	4.8	169	4.8	405	11.4	n.d	n.d
Potassium	1140	29.2	1350	34.6	129	3.3	174	4.5	562	14.4	568 ± 513	14.6 ± 13.2
Calcium	311	7.8	250	6.3	86	2.2	60	1.5	173	4.3	n.d	n.d
Magnesium	259	10.7	130	5.3	59	2.4	42	1.7	130	5.3	n.d	n.d
Sodium	366	15.9	276	12	128	5.6	123	5.3	112	4.9	n.d	n.d
Iron	3.11	56	2.03	36	2.02	36	1.42	25	1.4	25	n.d	n.d
Manganese	1.83	33.3	0.94	17.1	0.93	16.8	0.89	16.3	1.47	26.8	n.d	n.d
**Parameter**	µg L^−1^	µM	µg L^−1^	µM	µg L^−1^	µM	µg L^−1^	µM	µg L^−1^	µM	µg L^−1^	µM
Copper	110	1.7	54.7	0.9	117	1.8	<20	<0.3	55.8	0.9	n.d	n.d
Nickel	56.3	1	14.5	0.3	11.7	0.2	6.9	0.1	7.9	0.1	n.d	n.d
Zinc	194	3	174	2.7	182	2.8	81	1.2	136	2.1	n.d	n.d
pH	7.2		7		6.5		7.5		7		n.d	
Dry Matter	1.10%		1.13%		0.30%		0.23%		0.49%		0.5% ± 0.52%	

* Standard deviation; ^1^ Orthophosphate; ^2^ Molybdate Reactive Phosphate; ^3^ Minogue et al. [14]; n.d—Not Determined; COD—Chemical Oxygen Demand; BOD_5_—Five Day Biochemical Oxygen Demand; TON—Total Oxidised Nitrogen.

**Table 2:** Chemical composition of the 10% synthetic dairy soiled water based on Sample 1.

Chemical	Chemical Formula	Concentration	
		**mM**	**mg L^−1^**
Magnesium sulphate heptahydrate	(MgSO_4_)7H_2_O	1.079	265.4
Potassium acetate	CH_3_CO_2_K	2.2	215.6
Ammonium acetate	NH_4_CH_3_CO_2_	1.3	100.1
Calcium sulphate	CaSO_4_	0.707	96.2
Sodium chloride	NaCl	1.52	88.2
Ammonium chloride	NH_4_Cl	1.6	84.8
Tripotassium phosphate	K_3_PO_4_	0.21	44.5
Ammonium sulphate	(NH_4_)_2_SO_4_	0.32	42.2
Calcium chloride	CaCl_2_	0.07	7.8
Potassium chloride	KCl	0.08	6.0
Iron (III) chloride	FeCl_3_	0.0057	0.923
Manganese (II) chloride tetrahydrate	(MnCl_2_)4H_2_O	0.0033	0.653
Zinc chloride	ZnCl_2_	0.0003	0.041
Copper (II) chloride dihydrate	(Cl_2_Cu)2H_2_O	0.00017	0.029
Nickel (II) chloride hexahydrate	(NiCl_2_)6H_2_O	0.000095	0.023

## Data Availability

The data presented in this study are available on request from the corresponding author.
